# Hemodynamic Instability Induced by Superselective Angiography of the Ophthalmic Artery

**DOI:** 10.1155/2013/408670

**Published:** 2013-03-19

**Authors:** Stephan Klumpp, Lydia M. Jorge, Mohammed Ali Aziz-Sultan

**Affiliations:** ^1^Department of Clinical Anesthesia, Jackson Memorial Hospital, University of Miami, Miami, FL 33136, USA; ^2^Department of Clinical Neurologic Surgery, 201 Pope Life Center, University of Miami, Miami, FL 33136, USA

## Abstract

Retinoblastoma is one of the most common ophthalmic neoplasms affecting children worldwide. Since its recent introduction, superselective ophthalmic artery injection of chemotherapy with melphalan has significantly reduced the need for enucleation in patients with advanced disease and also shown to have minimal adverse effects on visual acuity as compared to the conventional therapy. Although no severe complications resulting in strokes or deaths have been reported, this treatment modality is not without difficulties. In this case discussion, we describe an event that has occurred to several pediatric patients undergoing superselective angiography of the ophthalmic artery that may be due to an oculopulmonary type reflex causing significant hemodynamic instability and hypoxemia.

## 1. Introduction

Intra-arterial chemotherapy infusion of the ophthalmic artery is a relatively new endovascular procedure for the treatment of retinoblastoma and is always performed under general anesthesia [1, 2]. We describe a cardiovascular reflex in the pediatric retinoblastoma population during superselective angiography of the ophthalmic artery.

## 2. Case Presentation

This is an account of a typical response noted in multiple cases.

A two-year-old boy was diagnosed with retinoblastoma involving the right eye. Following consultation with the pediatric oncology and ophthalmology oncology services, the patient was referred for intra-arterial chemotherapy infusion of Melphalan to the right ophthalmic artery. General anesthesia was induced with Propofol 3 mg/kg, Midazolam 0.2 mg/kg, and Rocuronium 0.6 mg/kg. After induction, the patient was successfully intubated with a 4.5 cuffed endotracheal tube and pressure controlled ventilation was initiated. Sevoflurane (1.6 Vol%), oxygen (0.5 L/min), and air (1.5 L/min) were administered for maintenance of general anesthesia.

**Figure 1:**
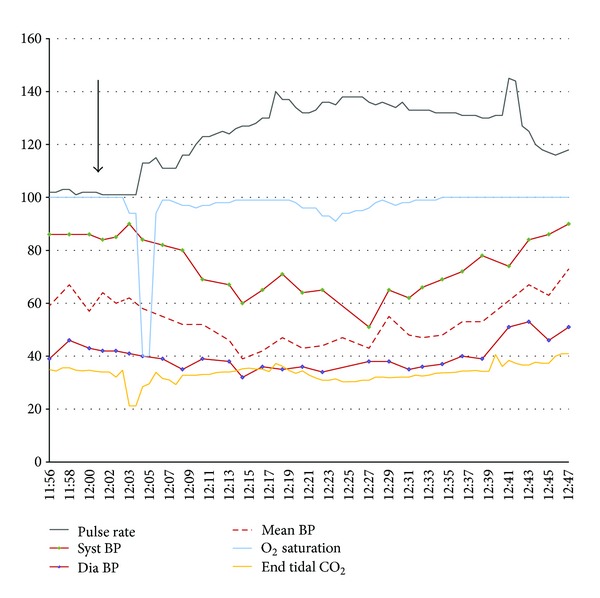
Vital signs recorded after stimulus occurred at 12:00.

Femoral access was obtained and a 4-French sheath was placed and perfused with heparinized saline. A 4-French Terumo angle glide catheter was navigated to the right internal carotid where cerebral angiograms were obtained. Under magnified roadmap guidance, a microcatheter was navigated into distal supraclinoid carotid. The microwire was withdrawn into the lumen of the microcatheter, and the microcatheter was slowly withdrawn within the carotid, thus selecting the ostium of the ophthalmic artery. Superselective angiography was performed demonstrating successful ophthalmic catheterization. Two minutes later, the patient's end tidal CO_2_ (EtCO_2_) decreased from 35 mmHg to 21 mmHg (normal 33–40 mmHg) followed by a subsequent deterioration in the oxygen saturation (SpO_2_) to 40% (normal 97%–100%). The microcatheter was withdrawn and ventilation with a FiO_2_ of 100% was initiated. On auscultation of the lungs, no abnormalities such as wheezing or rhonchi were appreciated. Within 4 minutes, the SpO_2_ and EtCO_2_ returned to baseline of 100% and 34–36 mmHg, respectively; however, the patient became hypotensive (Figure 1) requiring vasopressor support with Phenylephrine (2 mcg/kg) and fluid bolus for the next 30 minutes.

After stabilization, the procedure was resumed, the microcatheter was again navigated to the ophthalmic artery, and superselective angiography was performed. No further clinical changes were experienced. Manual micropulse infusion of chemotherapy proceeded over 30 minutes. Follow-up selective angiography demonstrated no change from baseline, and the microcatheter was removed. Final angiography demonstrated again no change from baseline, and the guide catheter was removed. After emergence from anesthesia, the patient was extubated and transported to the recovery room in stable condition. The patient was subsequently discharged home without any clinical sequela on postprocedure day 1.

## 3. Discussion

In our series of 30 pediatric patients with retinoblastoma, superselective ophthalmic microcatheterizations were performed 50 times under general anesthesia. We have experienced physiologic responses similar to those described in this case report on 8 distinct occasions. When elicited, the reflex unfolds as a characteristic and predictable sequence of events, which may vary in degree of magnitude. Severe manifestations of this phenomenon are observed as a temporary decrease in EtCO_2_ (mean decrease of EtCO_2_: 15.13 mmHg, range: 22 mmHg, and median: 13 mmHg) with profound hypoxia (mean decrease of SpO_2_ : 24.8 percentage points, range: 37 points, and median: 9.5 points) followed by prolonged hemodynamic instability with a decrease in blood pressure of over 50% from baseline. The mean time of the prolonged instability was 13 minutes (min) with a range of 37 min and median of 13 min. Milder forms are characterized by a decrease in EtCO_2_ with a concomitant decrease in SpO_2_ and minimal hemodynamic changes lasting less than 5 minutes.

This response is often accompanied by tachycardia. Bradycardia was never observed. The symptoms are triggered by one of two types of mechanical stimulation involving the ophthalmic artery: selective ophthalmic microcatheterization (the advancement of the catheter in the ophthalmic artery) or superselective ophthalmic angiography (the administration of contrast into the ophthalmic artery after catheter positioning). When elicited, the reaction occurs predictably within minutes after the stimulus. We have never seen the reflex start more than 5 minutes after the stimulus. Elicitation of the response appears isolated to the initial instance of offending mechanical stimulus. That is, if the reflex is not triggered by the initial microcatheterization or the initial selective angiography, in our experience, the reflex will not occur during the remainder of the procedure. Further, the reflex displays tachyphylaxis and has never occurred more than once during the same treatment session. These observations suggest a possible habituation mechanism. Three of the patients experienced the response on follow-up treatments (2 in the first month and 1 in the second month period). However, the magnitude of the response may vary.

In our series, no patient demonstrating the reflex experienced subsequent morbidity. In all instances, the patients were successfully stabilized and the procedures were resumed and carried to completion. All patients returned to baseline, and we have not experienced the need to alter routine postoperative management prescribed in the treatment protocol. When this phenomenon is experienced, the family is informed of the event.

The specific eliciting stimuli, predictable onset, stereotypical sequence of physiologic changes, and the possibility of habituation may suggest a reflex mechanism which may lead to an acute increase of pulmonary arterial pressure and of pulmonary vascular resistance. The ensuing elevation of the right ventricular afterload may lead to a decrease of right ventricular stroke volume and a decrease in cardiac output and blood pressure. The observed deterioration in lung compliance combined with the decrease in cardiac output results in the decreased EtCO_2_ and SpO_2_ due to decreased minute ventilation and an increased right to left pulmonary shunt. The precise nature of the physiologic process that triggers the described reflex is unclear.

An allergic reaction must be considered in any patients experiencing hypotension after injection of contrast media [3]. The incidence of acute allergic type reaction to nonionic iodinated contrast material has been documented approximately 0.18% in the pediatric population [4]. Anaphylaxis or anaphylactoid reaction appears unlikely, since the contrast media were given prior to and after entering the ophthalmic artery and in subsequent treatments without eliciting any reactions. In one patient, the reflex was elicited by mechanical stimulation during advancement of the catheter into the ophthalmic artery without the administration of contrast at this time.

The oculocardiac reflex has been well known in the literature for over 50 years [5]. More recently, the reflex has been identified as occurring during select endovascular procedures [6]. We described an activation of a similar reflex in the pediatric retinoblastoma population during selective angiography of the ophthalmic artery. However, the patients in our series did not show the typical bradycardia of the oculocardiac reflex.

## 4. Conclusion

The symptoms described may represent an acute onset of pulmonary hypertension mediated by release of vasoactive hormones or secondary to a vagal response triggered by stimulation of the ophthalmic artery. However, further investigations are needed to improve the understanding of the manifestations, management, and clinical significance of the described oculopulmonary reflex.
